# Relating hearing loss and executive functions to hearing aid users' preference for, and speech recognition with, different combinations of binaural noise reduction and microphone directionality

**DOI:** 10.3389/fnins.2014.00391

**Published:** 2014-12-04

**Authors:** Tobias Neher

**Affiliations:** Medical Physics and Cluster of Excellence Hearing4all, Oldenburg UniversityGermany

**Keywords:** hearing loss, cognition, hearing aids, signal processing, individual differences, personalized treatment

## Abstract

Knowledge of how executive functions relate to preferred hearing aid (HA) processing is sparse and seemingly inconsistent with related knowledge for speech recognition outcomes. This study thus aimed to find out if (1) performance on a measure of reading span (RS) is related to preferred binaural noise reduction (NR) strength, (2) similar relations exist for two different, non-verbal measures of executive function, (3) pure-tone average hearing loss (PTA), signal-to-noise ratio (SNR), and microphone directionality (DIR) also influence preferred NR strength, and (4) preference and speech recognition outcomes are similar. Sixty elderly HA users took part. Six HA conditions consisting of omnidirectional or cardioid microphones followed by inactive, moderate, or strong binaural NR as well as linear amplification were tested. Outcome was assessed at fixed SNRs using headphone simulations of a frontal target talker in a busy cafeteria. Analyses showed positive effects of active NR and DIR on preference, and negative and positive effects of, respectively, strong NR and DIR on speech recognition. Also, while moderate NR was the most preferred NR setting overall, preference for strong NR increased with SNR. No relation between RS and preference was found. However, larger PTA was related to weaker preference for inactive NR and stronger preference for strong NR for both microphone modes. Equivalent (but weaker) relations between worse performance on one non-verbal measure of executive function and the HA conditions without DIR were found. For speech recognition, there were relations between HA condition, PTA, and RS, but their pattern differed from that for preference. Altogether, these results indicate that, while moderate NR works well in general, a notable proportion of HA users prefer stronger NR. Furthermore, PTA and executive functions can account for some of the variability in preference for, and speech recognition with, different binaural NR and DIR settings.

## Introduction

Substantial variability in outcome is a consistent finding in hearing aid (HA) research. This holds true for a broad range of HA technologies, including amplification (e.g., Gatehouse et al., 2006a,b), noise reduction (NR) processing (e.g., Lunner, 2003; Brons et al., 2013), microphone directionality (DIR; e.g., Ricketts and Mueller, 2000; Keidser et al., 2013), and frequency compression (e.g., Glista et al., 2009; Souza et al., 2013). Presumably, this variability is related to the fact that HA users can differ in terms of a multitude of peripheral, central-auditory, or cognitive characteristics, even if they have similar audiograms and ages (cf., CHABA, 1988). Consequently, it is of interest to identify associations between such user characteristics and HA users' response to different forms of HA processing, as this would enable the development of fitting rationales that can take these dependencies into account. This would then allow for more individualized HA fittings.

Generally speaking, however, knowledge of such associations is rather sparse. This holds true especially for HA technology other than amplification. What is more, findings from related research studies are not always easily reconcilable with each other. One case in point is the role that executive functions play for benefit from different types of HA processing. “Executive functions” is an umbrella term that is typically thought to encompass a diverse, but related and overlapping, set of cognitive abilities such as working memory, attention, inhibition, and mental flexibility (e.g., Chan et al., 2008). More recently, HA researchers have focused on how one of these abilities—working memory—impacts hearing-impaired listeners' response to different HA processing, including dynamic range compression, NR processing, and frequency compression. Taken together, these studies suggest that HA users with smaller working memory capacity fare better with less aggressive HA processing whereas HA users with larger working memory capacity fare better with more aggressive HA processing (e.g., Lunner and Sundewall-Thorén, 2007; Arehart et al., 2013; Ng et al., 2013). In these studies, working memory capacity was typically assessed using a measure of reading span (RS) after Daneman and Carpenter (1980), while HA outcome was typically assessed using objective (e.g., speech recognition) measures.

In two previous studies, we also investigated the influence of RS on response to NR processing (Neher et al., 2013, 2014). In addition to RS, we controlled PTA by testing four age-matched groups of elderly hearing-impaired listeners exhibiting either smaller (“H+”) or larger (“H−”) PTA and either longer (“C+”) or shorter (“C−”) RS. In terms of HA processing, we used a binaural NR algorithm and varied its strength from inactive through moderate to strong. In terms of assessing outcome, we collected objective (e.g., speech recognition) and subjective (i.e., overall preference) data at fixed signal-to-noise ratios (SNRs) between −4 and 8 dB. For the objective outcomes, we found little evidence that RS and PTA modulate NR outcome. For overall preference, on the other hand, we found that C− listeners preferred strong over moderate NR despite poorer speech recognition due to greater speech distortion, whereas C+ listeners did not. These differences could indicate that C− listeners are more affected by noise than C+ listeners and therefore favor greater noise removal (even at the expense of added speech distortions), whereas C+ listeners prioritize fewer speech distortions.

The fact that we could only see a clear influence of RS in our preference data and that poorer RS was associated with preference for stronger NR is in contrast to the findings summarized above basically suggesting the opposite data pattern for objective HA outcomes. In view of this discrepancy and the general shortage of research dealing with relations between executive functions and subjective HA outcome, we wanted to scrutinize the influence of RS on preferred NR strength. In addition, we wanted to investigate the influence of PTA and input SNR. This aim was motivated by indications in our previous data (see Table 2 in Neher et al., 2013) that preference for strong NR increases with input SNR (mean preference for strong NR across listener groups: 40, 46, 51, and 57% at −4, 0, 4, and 8 dB SNR, respectively) and that H− listeners prefer stronger NR than H+ listeners (mean preference for inactive, moderate, and strong NR across SNRs: 5, 44, and 51% for H− listeners and 10, 44, and 46% for H+ listeners, respectively). Furthermore, we wanted to investigate the influence of preprocessing our stimuli with a directional microphone. Because it attenuates non-frontal signal components and thus their impact on the NR gains computed for, and applied to, the signal mixture, a forward-facing directional microphone can reduce the amount of distortion in a frontal speech signal (cf., Neher et al., 2014). Given that recent research has linked executive functions to susceptibility to distortion caused by HA processing (Lunner et al., 2009; Arehart et al., 2013), it is possible that less speech distortion due to directional preprocessing leads to stronger preference for strong NR, at least for HA users with certain cognitive profiles. Moreover, we wanted to determine if any associations between HA outcome and RS are also apparent for other measures of executive function. Previous research has shown that different measures of executive function are not necessarily strongly correlated (e.g., Gatehouse and Akeroyd, 2008; Neher et al., 2012), suggesting at least partially independent executive processes. It is therefore possible that different measures of executive function are related differently to HA outcome (e.g., that while shorter RS is related to greater benefit from less NR, poorer performance on another measure of executive function might be related to greater benefit from more NR). To address this possibility we included two additional measures of executive function. That is, we selected two visual measures that (1) were non-verbal in nature, (2) were designed to tap into other executive functions than the (verbal) RS measure, and (3) differed from each other in terms of the range of executive functions covered (broader vs. narrower). Our rationale for doing so was to find out if these relatively different measures would give rise to similar patterns of association with listeners' response to our HA conditions. Finally, to address the apparent discrepancy between objective and subjective HA outcomes alluded to above, we also measured speech intelligibility to find out if preference for, and speech recognition with, the different HA conditions are differentially related to PTA and executive functions.

In summary, the aims of the current study were to (1) replicate the previously observed association between RS and preferred binaural NR strength, (2) find out if the other measures of executive function give rise to similar patterns of association, (3) determine if PTA, input SNR, and DIR also modulate preferred NR strength, and (4) find out if for speech recognition results are similar. Due to the lack of comparable research, the current study was rather exploratory in nature. Nevertheless, based on the results summarized above we hypothesized that (1) poorer RS and larger PTA would be associated with stronger preference for strong NR, (2) RS and the other measures of executive function would be differentially related to preference for HA processing, (3) preference for strong NR would increase with input SNR, DIR would reduce the amount of speech distortion and thus potentially weaken any observed relations between preferred NR strength and the measures of executive function, and (4) the associations between speech recognition, PTA, and the measures of executive function would be different from those for preference.

## Materials and methods

Ethical approval for all experimental procedures was obtained from the ethics committee of the University of Oldenburg.

### Participants

Participants were recruited from a cohort of several hundred hearing-impaired listeners belonging to the database of the Hörzentrum Oldenburg, Germany. Selection criteria were bilateral sensorineural hearing losses, asymmetry in air-conduction thresholds of no more than 15 dB HL across ears for the standard audiometric frequencies from 0.5 to 4 kHz, and air-bone gaps of no larger than 15 dB HL at any audiometric frequency between 0.5 and 4 kHz. Furthermore, all participants were required to be habitual HA users with at least 9 months of HA experience, to have normal or corrected-to-normal vision according to the Snellen eye chart (i.e., 20/40 acuity or better), to have no history of any psychiatric disorders (e.g., depression), and to have a DemTect score of at least 9 (with a score of 8 being the cutoff point for suspected dementia; Kalbe et al., 2004). Initially, we selected 120 participants who satisfied these criteria and administered the RS measure (see below) to them. For further testing, we then selected 60 participants whom we could stratify into four well-matched groups based on the medians of their PTA and RS data. This (“H±C±”) approach was consistent with our previous studies except that we increased the sample size from 40 to 60 participants this time to allow us to investigate the effects of interest more fully. None of these participants had taken part previously. However, most of them had experience with similar research studies. Participants were paid on an hourly basis for their participation.

Table 1 summarizes the main characteristics for all 60 participants and the H+C+, H+C−, H−C+, and H−C− subgroups. Performing one-way analyses of variance (ANOVAs) with Bonferroni *post-hoc* analyses on the age, PTA, and RS data of these subgroups confirmed (1) the lack of significant differences in terms of age [*F*_(3, 56)_ = 0.4; *p* > 0.7; η^2^_*p*_ = 0.02], (2) significant differences in terms of PTA [*F*_(3, 56)_ = 40.0; *p* < 0.0001, η^2^_*p*_ = 0.68] between all pairs of subgroups with different hearing status (all *p* < 0.0001) but no significant difference in terms of PTA between any two subgroups with the same hearing status (all *p* = 1.0), and (3) significant differences in terms of RS [*F*_(3, 56)_ = 41.7; *p* < 0.0001, η^2^_*p*_ = 0.69] between all pairs of subgroups with different RS status (all *p* < 0.0001) but no significant difference in terms of RS between any two subgroups with the same RS status (all *p* = 1.0). Compared to the cohort we had tested previously, these participants had slightly lower age (all subgroups), slightly better RS (all subgroups), and slightly smaller PTA (H− subgroups).

**Table 1:** **Means (and ranges) for the age, PTA, RS, and EC_PC_ data of all 60 participants as well as the H+C+, H+C−, H−C+, and H−C− subgroups (*N* = 15 per subgroup)**.

	**Age (yr)**	**PTA (dB HL)**	**RS (%-correct)**	**EC_PC_ (%-correct)**
All participants	72 (60–82)	45 (28–67)	38 (19–57)	86 (35–100)
H+C+	73 (61–81)	36 (28–45)	46 (39–56)	88 (35–100)
H−C+	71 (64–82)	54 (46–67)	47 (39–57)	76 (53–100)
H+C−	72 (63–80)	38 (29–45)	30 (19–37)	90 (73–100)
H−C−	73 (60–81)	52 (46–62)	30 (22–35)	90 (78–100)

### Measures of executive function

To assess executive function we used the RS measure after Daneman and Carpenter (1980) and two subtests from the commercially available, clinically validated “TAP-M” test battery (Zimmermann and Fimm, 2012). The TAP-M test battery was developed to assess elderly persons in terms of fitness for driving. The two measures used here were the so-called “distractibility” (DIS) and “executive control” (EC) subtests.

#### Reading span (RS) measure

The RS measure is a visual, verbal measure of working memory capacity that is rather widely used in audiological research (e.g., Neher et al., 2011, 2013; Arehart et al., 2013; Desjardins and Doherty, 2013; Ng et al., 2013). Our implementation, which is based on psycholinguistically controlled test items, closely mimics that of other researchers (cf., Carroll et al., 2014). It consists of a training round comprising three trials (which we carried out as often as needed until the participant had understood the task) and a test round comprising 54 trials (which we carried out once). On each trial, short sentence segments are displayed on a screen one at a time at a rate of one word per 0.8 s. After three segments, there is a pause of 1.75 s, during which the participant has to respond either “yes” if the previous three segments made up a semantically correct sentence (e.g., “Das Mädchen–sang–ein Lied”; “The girl–sang–a song”) or “no” if the previous three segments made up a semantically absurd sentence (e.g., “Die Flasche–trank–Wasser”; “The bottle–drank–water”). Following a sequence of sentences (three, four, five, or six, in random order), the participant is asked to recall either the first or final words of all the three, four, five, or six previous sentences in any order. As before, we used the percentage of correctly recalled first and final words presented across the 54 trials to assess performance.

#### Distractibility (DIS) measure

The DIS subtest from the TAP-M test battery is a visual, non-verbal measure of executive function, which according to its developers taps into selective attention and inhibition (Zimmermann and Fimm, 2012). In the middle of a computer screen, happy or sad smiley symbols are presented for short instances of time. The participant has to respond as quickly as possible by pressing a button whenever a sad smiley appears, but not when a happy smiley appears. At irregular timing intervals, distractor stimuli (i.e., abstract shapes or symbols) appear somewhere near the middle of the screen. These distractors are colored to make them perceptually more salient than the smileys, which are shown in black and white only. The DIS measure consists of a training round comprising 11 trials (which we carried out as often as needed until the participant had understood the task) and a test round comprising 150 trials (which we carried out once). In the test round, 60 target smileys are presented, 30 of which are preceded by a distractor. On average, the (randomized) duration of a trial is 2.3 s. The distractor and target stimuli are separated in time by 0.5 s. Distractors remain on the screen for 1.5 s, while target stimuli are only visible for 0.15 s. In accordance with recommendations given in the TAP-M manual we decided to explore two DIS performance measures: (1) the difference in median response time between correctly responded to target stimuli with and without preceding distractors (“DIS_ΔRT_”), and (2) the difference in the proportion of correct responses (calculated by subtracting the number of missed targets and wrong responses from 30 and dividing the result by 30) between trials with and without preceding distractors.

#### Executive control (EC) measure

The EC subtest from the TAP-M test battery is a visual, non-verbal measure of executive function, which according to its developers taps into working memory, mental flexibility, selective attention, and inhibition (Zimmermann and Fimm, 2012). In the middle of a computer screen, red or blue numbers and letters are presented one at a time for 0.5 s. The participant has to respond as quickly as possible to red numbers by pressing a left button and to blue letters by pressing a right button, and to ignore blue numbers and red letters. The EC measure consists of a training round comprising 10 trials with five target stimuli (which we carried out as often as needed until the participant had understood the task) and a test round comprising 80 trials with 40 target stimuli (which we carried out once). The timing interval between consecutive stimuli varies randomly between 2 and 3 s. In accordance with recommendations given in the TAP-M manual we decided to explore two EC performance measures: (1) the median response time to correctly responded to target stimuli (“EC_RT_”), and (2) the proportion of correct responses calculated by subtracting the number of missed targets and wrong responses from 40 and dividing the result by 40 (“EC_PC_”). Despite several training rounds one participant was unable to carry out this test successfully, so we abandoned it in his case.

### Physical test setup

The auditory tests were carried out in a soundproof booth. Inside the booth two computer screens were located. One screen was used for displaying information to the participants. The other screen, which the participants were unable to see, was used by an experimenter for scoring the participants' responses during the speech recognition measurements. All test software was implemented in MatLab (MathWorks, Natick, USA). Audio playback was via an Auritec (Hamburg, Germany) Earbox Highpower soundcard and a pair of Sennheiser (Wennebostel, Germany) HDA200 headphones. Calibration was carried out using a Brüel & Kjær (B&K; Nærum, Denmark) 4153 artificial ear, a B&K 4134 1/2″ microphone, a B&K 2669 preamplifier, and a B&K 2610 measurement amplifier.

The RS, DIS, and EC measures were administered in a quiet well-lit room. A computer screen displaying the stimuli was positioned about 0.5 m in front of the participants' face. During the DIS and EC measurements, participants responded to the stimuli using two large hardware buttons supplied with the TAP-M test battery.

### Speech stimuli

The speech stimuli closely resembled those from our previous studies. They were based on recordings from the Oldenburg sentence material (Wagener et al., 1999), which consists of 120 sentences that are low in semantic context and that all follow the form “name verb numeral adjective object” (e.g., “Thomas has two large flowers”). To simulate a realistic complex listening situation we convolved the sentence recordings with pairs of head-related impulse responses (HRIRs). These HRIRs were measured in a large, reverberant cafeteria using a B&K head-and-torso simulator (HATS) equipped with two three-microphone behind-the-ear Siemens Acuris HA “dummies” (Kayser et al., 2009). Each dummy consisted of the microphone array housed in its original casing, but without any of the integrated amplifiers, speakers, or signal processors commonly used in HAs. For the purpose of the current study, we used HRIRs measured with the front and rear (but not the mid) microphones and a frontal source at a distance of 1 m from, and at the same height as, the HATS. Following convolution with these HRIRs, the speech signals ranged in length from 2.2 to 3.2 s. For the interfering signal, we used a recording made in the same cafeteria with the same setup during a busy lunch hour. On each trial, a 5-s extract from this recording was randomly chosen and processed to have 50-ms raised-cosine on- and offset ramps. The resultant signal was presented at a nominal sound pressure level of 65 dB. It was mixed with a given target sentence, which started 1.25 s after the cafeteria noise and which was adjusted in level to produce a given SNR.

### Hearing aid processing

All signal processing was implemented on the Master Hearing Aid (MHA) research platform of Grimm et al. (2006). It included DIR, binaural NR, linear amplification, and headphone equalization and was carried out at a sampling rate of 16 kHz. Before presentation, stimuli were resampled to 44.1 kHz. A total of six HA conditions were tested, which we will refer to as DIR_off_NR_off_, DIR_off_NR_mod_, DIR_off_NR_str_, DIR_on_NR_off_, DIR_on_NR_mod_, and DIR_on_NR_str_. These conditions differed in terms of whether (1) pairs of omnidirectional (“DIR_off_”) or cardioid (“DIR_on_”) microphones were used and (2) the binaural NR scheme was set to inactive (“NR_off_”), moderate (“NR_mod_”), or strong (“NR_str_”) processing.

#### Microphone directionality (DIR)

To simulate a pair of omnidirectional microphones we used the speech and noise signals obtained through convolution with the HRIRs measured with the front microphones of the two HA dummies. To simulate two directional microphones we employed the speech and noise signals obtained through convolution with the HRIRs measured with the front and rear microphones of the two HA dummies. Using a simple delay-and-sum beamformer algorithm (Elko and Pong, 1995), we then processed the two microphone signals per HA dummy in such a way that we obtained a pair of static forward-facing cardioid microphones. To compensate for the high-pass characteristic that is typical of directional microphones (e.g., Dillon, 2012), we applied a 1024th-order finite impulse response (FIR) filter to the output of each cardioid microphone. This filter ensured that the cardioid microphones were matched in terms of frequency response to their omnidirectional counterparts for the frontal (0° azimuth) source direction. We then also applied a two-channel 1024th-order FIR filter to the left and right outputs of each pair of omnidirectional or cardioid microphones. This filter ensured that the pairs of omnidirectional and cardioid microphones were matched in terms of their interaural phase and level differences for the frontal (0° azimuth) source direction. Directional microphone arrays are very sensitive to inter-microphone mismatch, which can result in considerable distortion of interaural cues (Van Den Bogaert et al., 2005). Thus, by post-processing the microphone signals in this manner, we made sure that the frontal target signals of our stimuli sounded highly similar across the omnidirectional and cardioid settings.

#### Binaural noise reduction (NR)

The binaural NR scheme was identical to that from our previous study (see Neher et al., 2013 for details). In short, it consisted of a Fast Fourier Transform-based filterbank with 12 frequency bands covering an 8-kHz bandwidth. Using a 40-ms integration time constant, the binaural coherence (or interaural similarity) of the left and right input signals is first estimated in each frequency band. These estimates can take on values between 0 and 1. A value of 0 corresponds to fully incoherent (or diffuse) sound, while a value of 1 corresponds to fully coherent (or directional) sound. Because of diffraction effects around the head, the binaural coherence is always high below about 1 kHz. At higher frequencies, the coherence is low for diffuse and reverberant signal components, but high for the direct sound from nearby sources. Due to the spectro-temporal fluctuations contained in speech, the ratio between (undesired) incoherent and (desired) coherent signal components may vary across time and frequency. By applying appropriate time- and frequency-dependent gains this ratio can be improved. These gains are derived by applying an exponent, α, to the coherence estimates. As in our previous study, we tested three values of α: 0, 0.75, and 2. In this manner, we could vary the NR strength from inactive (α = 0) through moderate (α = 0.75) to strong (α = 2).

#### Linear amplification and headphone equalization

To ensure adequate audibility we spectrally shaped all speech stimuli according to the National Acoustic Laboratories-Revised Profound (NAL-RP) prescription rule (Byrne et al., 1991). Specifically, for each participant we determined the required gain at 250, 500, 1000, 1500, 2000, 3000, 4000, and 6000 Hz and mapped the resultant values onto the MHA filterbank using interpolation techniques. Finally, we processed the left and right channels of each stimulus with a 32nd-order FIR filter that compensated for the uneven magnitude response of the headphones.

#### Physical effects

**Figure 1:**
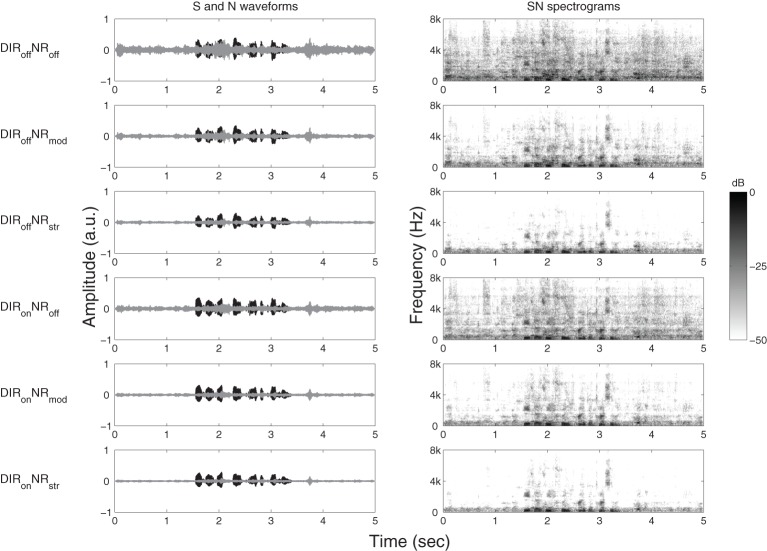
**Graphical illustration of the effects of DIR_off_NR_off_, DIR_off_NR_mod_, DIR_off_NR_str_, DIR_on_NR_off_, DIR_on_NR_mod_, and DIR_on_NR_str_ processing on (one channel of) an example stimulus with an input SNR of 4 dB**. Panels on the left-hand side show time waveforms of the target speech, S (black) and the cafeteria noise, N (gray). Panels on the right-hand side show corresponding spectrograms for the signal mixtures, SN. a.u. denotes arbitrary units.

The chosen HA conditions gave rise to a number of physical effects, which are illustrated in Figure 1 for one channel of an example stimulus with an input SNR of 4 dB. The panels on the left-hand side show, for each HA condition, the waveforms of the speech and noise signals at the output of the simulated HA. The panels on the right-hand side show the spectrograms of the signal mixtures. The dominant effect of moderate and especially strong NR is to suppress incoherent signal components above about 1 kHz. To quantify the physical effects of our HA conditions we calculated the speech-weighted SNR improvement (“ΔAI-SNR”) for input SNRs of −4, 0, and 4 dB using a 2-min speech-in-noise stimulus. That is, we first estimated the SNR improvement relative to DIR_off_NR_off_ in one-third octave bands and then took the scalar product of these estimates and the one-third octave band importance function from the Speech Intelligibility Index (ANSI, 1997). Table 2 summarizes the results. Relative to the omnidirectional setting, the cardioid setting led to a ΔAI-SNR of 3.3 dB, irrespective of input SNR. Furthermore, ΔAI-SNR increased with NR strength (e.g., from 1.7 dB for DIR_off_NR_mod_ to 2.8 dB for DIR_off_NR_str_ at 0 dB SNR) and input SNR (e.g., from 1.5 dB at −4 dB SNR to 3.8 dB at 4 dB SNR for DIR_off_NR_str_). It is also worth noting that, with the cardioid setting, the ΔAI-SNRs brought about by moderate and strong NR increased by, respectively, 0.3 and 0.6 dB at −4 dB SNR and by, respectively, 0.2 and 0.3 dB at 0 dB SNR; at 4 dB SNR, microphone mode basically had no influence on the ΔAI-SNRs due to moderate and strong NR.

**Table 2:** **Speech-weighted SNR improvement (ΔAI-SNR) relative to DIR_off_NR_off_ for DIR_off_NR_mod_, DIR_off_NR_str_, DIR_on_NR_off_, DIR_on_NR_mod_, and DIR_on_NR_str_ and input SNRs of −4, 0, and 4 dB**.

**HA condition**	**ΔAI-SNR (dB)**		
	**−4 dB SNR**	**0 dB SNR**	**4 dB SNR**
DIR_off_NR_mod_	1.0	1.7	2.3
DIR_off_NR_str_	1.5	2.8	3.8
DIR_on_NR_off_	3.3	3.3	3.3
DIR_on_NR_mod_	4.6	5.1	5.5
DIR_on_NR_str_	5.4	6.4	7.0

In addition to SNR improvement, we quantified the amount of speech distortion caused by our HA conditions. To that end, we analyzed the stimuli from the ΔAI-SNR calculations using the Hearing Aid Speech Quality Index (HASQI; Kates and Arehart, 2014). HASQI assesses the amount of signal degradation in a processed stimulus relative to an unprocessed reference stimulus. It returns a value between 0 and 1, with 0 indicating very low fidelity and 1 indicating perfect fidelity. Because we were interested in the adverse effects of NR, we used the inactive NR setting as reference for the moderate and strong NR settings. Because we were also interested in the effects of directional preprocessing we performed these analyses separately for the omnidirectional and cardioid settings. In each case, we analyzed the target speech signals processed with the NR gains computed for the corresponding signal mixtures.

The HASQI values that we obtained ranged from 0.59 for strong NR without directional preprocessing at −4 dB SNR to 0.88 for moderate NR with directional preprocessing at 4 dB SNR (see Table 3). As expected, signal fidelity increased with SNR (mean HASQI values across NR and DIR setting: 0.72, 0.75, and 0.78 for −4, 0, and 4 dB SNR, respectively) and decreased with NR strength (mean HASQI values across SNR and DIR setting: 0.85 and 0.65 for moderate and strong NR, respectively). Furthermore, directional preprocessing had a positive effect on signal fidelity (mean HASQI values across SNR and NR setting: 0.74 and 0.76 for DIR_off_ and DIR_on_, respectively). Altogether, these data show that the efficacy of our NR scheme increased with SNR, in terms of both SNR improvement and speech quality. Furthermore, not only did the cardioid setting lead to a considerable SNR improvement, it also reduced the speech distortion caused by moderate and strong NR.

**Table 3:** **Speech distortion (as measured using HASQI) caused by moderate and strong NR for the omnidirectional (DIR_off_) and cardioid (DIR_on_) settings and input SNRs of −4, 0, and 4 dB**.

**NR strength**	**Speech distortion (HASQI)**					
	**−4 dB SNR**		**0 dB SNR**		**4 dB SNR**	
	**DIR_off_**	**DIR_on_**	**DIR_off_**	**DIR_on_**	**DIR_off_**	**DIR_on_**
Moderate	0.82	0.85	0.84	0.86	0.86	0.88
Strong	0.59	0.63	0.64	0.67	0.68	0.71

### Speech recognition measurements

Consistent with our earlier studies, we determined speech recognition at −4 and 0 dB SNR. Since we had previously observed good test-retest reliability for similar measurements at these SNRs, we only made one measurement per condition. For the current study, we distributed the 12 measurements (6 HA conditions × 2 SNRs) in such a way that, at each of the two visits per participant (see Test protocol), three measurements per SNR were performed, each of the six HA conditions was tested once, and that the order of presentation was randomized. Furthermore, we started each visit with two training measurements carried out with DIR_on_NR_off_ processing at 4 and then 0 dB SNR. In total, each participant therefore completed 16 measurements. For each of these, we used a different test list (consisting of 20 five-word sentences each) and also balanced the lists across participants. Following the presentation of a stimulus, participants had to repeat the words they had understood, which an experimenter scored using a graphical user interface (GUI).

### Overall preference judgments

For the preference judgments, we asked our participants to imagine being inside the cafeteria and wanting to communicate with the speaker of the sentences. They then had to compare a given pair of HA settings and decide which one they preferred overall. In doing so, they were instructed to pay attention to both target speech and background noise. Test conditions were identical to the speech recognition measurements, except that we also tested at 4 dB SNR. On each trial, six 5-s stimuli were generated as described above and concatenated, resulting in a 30-s stimulus. Comparisons were blocked by SNR. Different (randomly selected) speech signals and noise extracts were used for the different SNRs. Using a GUI and a touch screen, participants controlled playback of the (looped) stimuli and entered their responses. Participants completed four or five rounds of preference judgments (see Test protocol). One round consisted of 45 pairwise comparisons (3 SNRs × 15 possible combinations of the six HA conditions) in randomized order. At the start of the first round, six trials were initially presented for training purposes at 0 dB SNR. Presentation of the HA conditions was balanced in that the order of allocation of a given pair of HA conditions to the two buttons controlling playback was switched from one round to the next (e.g., DIR_off_NR_off_ vs. DIR_off_NR_str_ in the first round and DIR_off_NR_str_ vs. DIR_off_NR_off_ in the second round). The different rounds were not exact retests, as all stimuli were newly generated at the beginning of a round.

### Test protocol

All participants attended two 1.5-h visits. Each visit started with the speech recognition measurements (ca. 25 min) followed by the preference judgments. At the first visit, each participant completed two rounds of preference judgments (ca. 20 min each). At the second visit, 35 participants completed another two rounds of preference judgments, while the other participants were able to complete three rounds each within the allotted time. After the speech recognition measurements and in-between the preference judgments participants were asked to take 5-min breaks.

### Statistical analyses

In preparation for the statistical analyses, we divided the speech scores by 100 and transformed them into rationalized arcsine units (RAU; Studebaker, 1985). Furthermore, we converted the preference judgments into scores ranging from 0 to 1 by calculating, for each SNR, the total number of times a given HA condition was preferred to the other five conditions and then dividing the result by the total number of comparisons per condition (e.g., David, 1963; Arehart et al., 2007; Anderson et al., 2009). To avoid the influence of extreme values on our results and to normalize the variance in our datasets we excluded scores more than three times the interquartile range away from the lower and upper quartiles of a given dataset. Thus, we removed the DIS data of two participants. Furthermore, we excluded one participant altogether as, despite belonging to the H+C+ group, her speech scores were extraordinarily poor (grand average speech recognition: 11% correct). Finally, we also arcsine-transformed the proportions of correct responses from the EC measure.

Next, we carried out regression analyses with the aim of identifying the most predictive sets of between-subject factors for the speech and preference scores. Consistent with the H±C± approach we had used previously, we dichotomized the chosen predictors using a median split. In this way, we obtained two subgroups (or mean scores) per predictor, one denoting better ability (e.g., smaller PTA or longer RS) and one denoting worse ability (e.g., larger PTA or shorter RS). In a few cases, individual scores were equivalent to the overall median of a given dataset and thus had to be excluded. Subgroups therefore differed in size, but in no case included fewer than 24 individual scores. To test for statistically significant differences among our experimental variables we then performed mixed-model ANOVAs. Whenever appropriate, we corrected for violations of sphericity using the Greenhouse-Geisser correction. Furthermore, we included age as a covariate in each model. To leave the within-subject factor sum of squares unaltered we first centered the age variable by subtracting the overall sample mean from each data point (cf., Fidell and Tabachnick, 2006; Van Breukelen and Van Dijk, 2007).

Because of differences in the way we measured speech recognition and in the way we analyzed the preference data between our previous and the current study, we did not have estimates of test-retest reliability available and thus could not perform any power analyses.

## Results

### Analysis and selection of between-subject factors

To identify the most effective predictors for the speech and preference scores we performed a series of multivariate multiple regression analyses. Using age as our baseline model, we assessed the predictive power of PTA and the measures of executive function both separately and in different combinations. In this manner, we could determine the unique variance explained by each predictor as well as the total variance explained by a given set of predictors. For each model tested, we averaged the explained variance across the various datasets per outcome (speech recognition: 2 SNRs × 6 HA conditions = 12 datasets; overall preference: 3 SNRs × 6 HA conditions = 18 datasets) to determine its total predictive power.

For the speech scores, we found that age accounted for 8.1% of the variance, while of the remaining predictors PTA, RS, DIS_ΔPC_, and DIS_ΔRT_ were most effective, accounting for 28.2, 13.5, 12, and 11%, respectively (together with age). The most effective combination consisted of PTA, RS, and DIS_ΔRT_ (unique *R*^2^: 20.1, 5.4, and 3.1%, respectively). Together with age, they accounted for 36.7% of the total variance in the speech scores (range across datasets: 30–46%).

For the preference scores, we found that age accounted for 3.5% of the variance, while of the remaining predictors PTA, EC_RT_, EC_PC_, and RS were most effective, accounting for 9.7, 6.2, 6.1, and 4%, respectively (together with age). The most effective combination consisted of PTA, EC_PC_, and EC_RT_ (unique *R*^2^: 6.1, 2.7, and 2.4%, respectively). Together with age, they accounted for 14.6% of the total variance in the preference scores. Closer inspection revealed that explained variance varied markedly across the 18 datasets (range: 1–27%). Predictive power was lower at −4 dB SNR (mean *R*^2^: 8%) than at 0 and 4 dB SNR (mean *R*^2^: 17 and 18%, respectively). Predictive power was also lower for the measurements made with moderate NR (mean *R*^2^: 9%) than for those made with inactive and strong NR (mean *R*^2^: 18 and 16%, respectively). For the measurements made with the omnidirectional and cardioid settings predictive power was similar (mean *R*^2^: 16 and 13%, respectively). It is also worth noting that, in contrast to our expectations, RS was an ineffective predictor of preferred HA condition. This will be discussed further below.

To complete the above analysis we computed pairwise Pearson's *r* correlation coefficients. The largest correlations that we found were the ones between RS and EC_PC_ and between PTA and EC_PC_, which were both rather weak (both *r* = 0.31, *p* = 0.02).

### Speech recognition

To further analyze the speech scores we performed an ANOVA with SNR and HA condition as within-subject factors, PTA, RS, and DIS_ΔRT_ as between-subject factors, and age as a covariate. Since we observed no statistically significant effects of DIS_ΔRT_ (i.e., the least predictive between-subject factor selected above) we removed it from the model. Table 4 provides a summary of the results. The effects of PTA and RS were statistically significant, as were the effects of SNR, HA condition, and SNR × HA condition. Furthermore, PTA interacted with HA condition, while for RS the two-way interaction with SNR and HA condition was significant.

**Table 4:** **Results from the ANOVA performed on the speech scores**.

**Model term**	***df***	***F***	***p***	**η^2^_*p*_**
**BETWEEN-SUBJECT FACTORS/COVARIATES**				
PTA	(1, 54)	14.7	<0.001	0.22
RS	(1, 54)	7.6	<0.01	0.12
Age	(1, 54)	8.7	<0.01	0.14
**WITHIN-SUBJECT FACTORS**				
SNR	(1, 54)	612	<0.00001	0.92
HA	(4.1, 221.1)	390	<0.00001	0.88
SNR × HA	(5, 270)	26.0	<0.00001	0.33
SNR × PTA	(1, 54)	6.4	<0.05	0.11
HA × PTA	(4.1, 221.1)	2.6	<0.05	0.05
SNR × HA × RS	(5, 270)	3.3	<0.01	0.06

*HA denotes HA condition. Model terms not shown were not statistically significant*.

**Figure 2:**
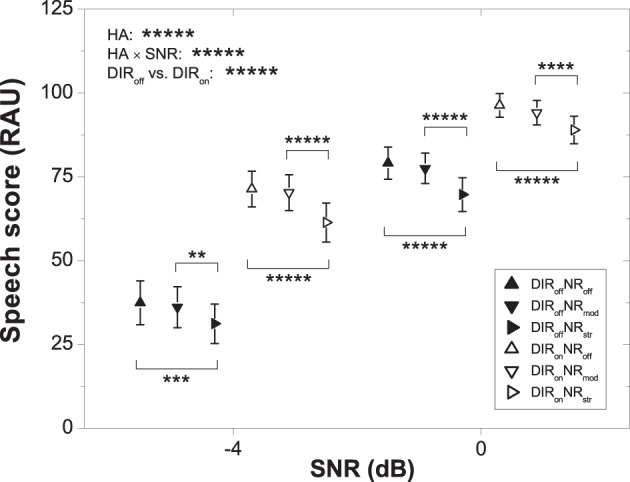
**Mean speech scores for the six HA conditions and two SNRs. Error bars show 95% confidence intervals**. Horizontal bars denote significant differences (^**^p < 0.01, ^***^p < 0.001, ^****^p < 0.0001, ^*****^p < 0.00001). HA denotes HA condition.

Figure 2 shows mean speech scores with 95% confidence intervals for the six HA conditions and two SNRs. As expected, speech recognition improved with SNR. To investigate the significant effect of HA condition further we carried out a series of planned contrasts. These revealed significant differences among all pairs of HA conditions (all *p* < 0.05) except for DIR_off_NR_off_ vs. DIR_off_NR_mod_ (*p* > 0.1). Thus, across the two SNRs moderate NR did not affect speech recognition when combined with the omnidirectional setting, whereas in combination with the cardioid setting it led to a reduction of about 1.5 RAU (*p* = 0.041). Furthermore, strong NR reduced speech recognition by about 7 RAU across the two SNRs irrespective of microphone mode, while relative to no DIR the cardioid setting improved speech recognition by about 25 RAU averaged across SNR and NR setting.

**Figure 3:**
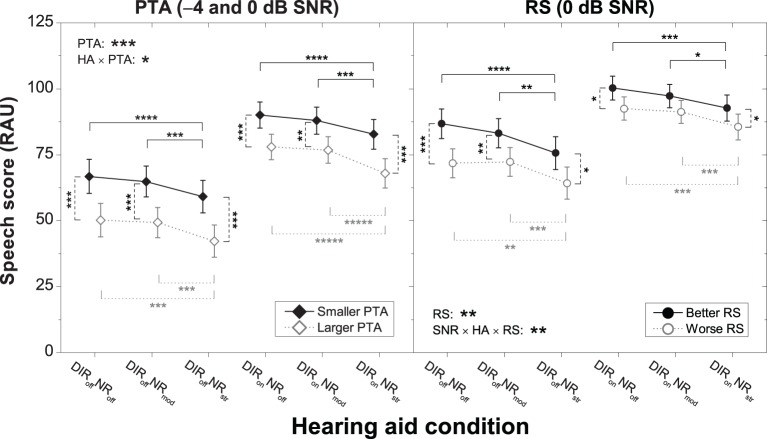
**Mean speech scores for the six HA conditions for the “smaller PTA” and “larger PTA” subgroups at −4 and 0 dB SNR (left panel) and the “better RS” and “worse RS” subgroups at 0 dB SNR (right panel)**. Error bars show 95% confidence intervals. Black solid horizontal bars denote significant differences for the “smaller PTA” and “better RS” subgroups, gray dotted horizontal bars denote significant differences for the “larger PTA” and “worse RS” subgroups, and black dashed vertical bars denote significant differences among pairs of subgroups for a given HA condition (^*^p < 0.05, ^**^p < 0.01, ^****^p < 0.0001, ^*****^p < 0.00001). HA denotes HA condition.

Figure 3 shows the speech scores of the two PTA subgroups (left panel) and the two RS subgroups (right panel) for the different HA conditions. As expected, the “smaller PTA” and “better RS” subgroups achieved better speech recognition than the “larger PTA” and “worse RS” subgroups. To investigate the significant interaction between PTA and HA condition further we carried out series of planned contrasts on the data from the “larger PTA” and “smaller PTA” subgroups. For the “smaller PTA” subgroup, we found that the decrement in speech recognition due to strong (relative to inactive) NR was basically unaffected by the microphone setting (7.7 vs. 7.3 RAU), whereas for the “larger PTA” subgroup it was slightly larger with the cardioid setting (8.0 vs. 10.0 RAU). These results suggest that in terms of speech recognition HA users with larger PTA fare slightly worse with strong NR than HA users with smaller PTA if the NR is applied in conjunction with a pair of directional microphones.

To investigate the significant two-way interaction between SNR, HA condition, and RS further we carried out separate ANOVAs on the data from −4 and 0 dB. We found that RS interacted with HA condition at 0 dB (*p* = 0.026) but not at −4 dB (*p* = 0.075). Thus, we analyzed the 0 dB data further by carrying out series of planned contrasts on the data from the “better RS” and “worse RS” subgroups. For the “worse RS” subgroup, we found that the decrement in speech recognition due to strong (relative to inactive) NR was basically unaffected by microphone setting (7.6 vs. 7.0 RAU), whereas for the “better RS” subgroup it was slightly larger with the omnidirectional setting (11.2 vs. 7.6 RAU). These results suggest that in terms of speech recognition HA users with larger RS fare slightly worse with strong NR than HA users with smaller RS if the NR is applied without directional microphones.

### Overall preference

Because the preference scores were proportional values reflecting how much a given HA condition was preferred to each of the other five HA conditions for a given SNR, we analyzed these scores further by performing three separate ANOVAs—one per SNR. In each model, we included HA condition as within-subject factor, PTA, EC_PC_, and EC_RT_ as between-subject factors, and age as a covariate. Since we observed no statistically significant effects of EC_RT_ (i.e., the least predictive between-subject factor selected above) we removed it from the models. Table 5 provides a summary of the results. For each SNR, we found a highly significant effect of HA condition. Furthermore, whereas neither PTA nor EC_PC_ interacted with HA condition at −4 dB, we found significant interactions between each of these factors and HA condition at 0 and 4 dB SNR. Table 1 therefore also provides a summary of the EC_PC_ data.

**Table 5:** **Results from the ANOVAs performed on the preference scores corresponding to −4, 0, and 4 dB SNR**.

**Model term**	**−4 dB SNR**				**0 dB SNR**				**4 dB SNR**			
	***df***	***F***	***p***	**η^2^_*p*_**	***df***	***F***	***p***	**η^2^_*p*_**	***df***	***F***	***p***	**η^2^_*p*_**
HA	(2.3, 108.2)	142	<0.00001	0.75	(1.8, 86.0)	71	<0.00001	0.60	(1.7, 81.0)	59	<0.00001	0.56
HA × PTA	(2.3, 108.2)	2.0	>0.1	0.04	(1.8, 86.0)	4.8	<0.05	0.09	(1.7, 81.0)	6.6	<0.01	0.12
HA × EC_PC_	(2.3, 108.2)	1.2	>0.2	0.03	(1.8, 86.0)	3.8	<0.05	0.07	(1.7, 81.0)	3.4	<0.05	0.07

*HA denotes HA condition. Model terms not shown were not statistically significant*.

**Figure 4:**
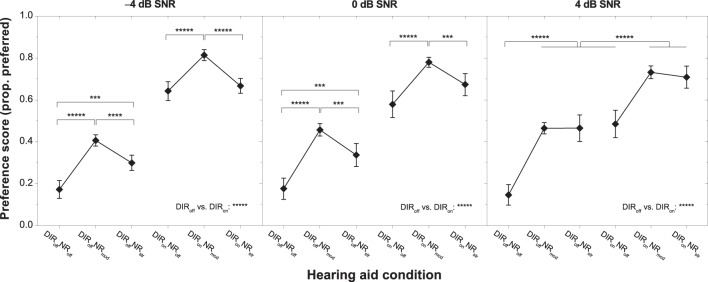
**Mean preference scores for the six HA conditions and SNRs of −4 dB (left panel), 0 dB (middle panel), and 4 dB (right panel)**. Error bars show 95% confidence intervals. Horizontal bars denote significant differences (^***^p < 0.001, ^****^p < 0.0001, ^*****^p < 0.00001).

Figure 4 shows the effect of HA condition on overall preference for each of the three SNRs tested. As already noted in the context of the regression analyses (see above), inter-individual variability in preferred NR strength was smallest for moderate NR and much larger for inactive and strong NR, especially at 0 and 4 dB SNR. To investigate the significant effect of HA condition further we carried out a series of planned contrasts on the data from each SNR. At −4 dB, we found that moderate NR was significantly preferred over inactive and strong NR with and without DIR (all *p* < 0.00001). Furthermore, we found that strong NR was significantly preferred over inactive NR without DIR (*p* < 0.01) but not over inactive NR with DIR (*p* > 0.7). At 0 dB, the pattern was very similar, although there was a tendency for preference for strong NR to increase, particularly so in combination with DIR. This trend continued at 4 dB such that moderate and strong NR were equally preferred both with and without DIR (both *p* > 0.3). In terms of directional benefit, we found a very strong preference for DIR over no DIR at all three SNRs (all *p* < 0.00001).

**Figure 5:**
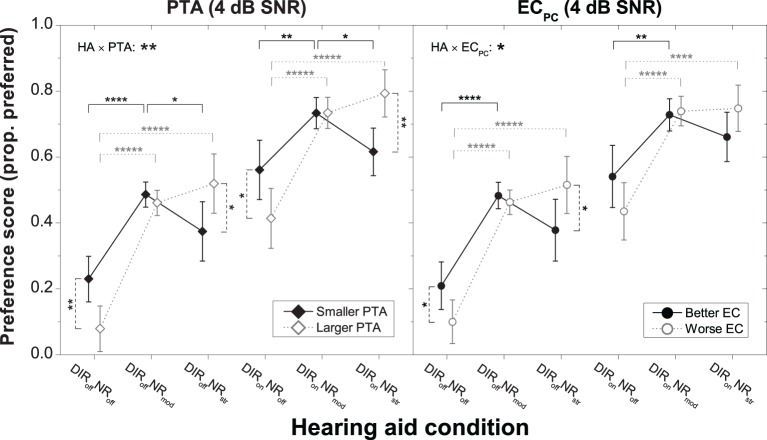
**Mean preference scores for the six HA conditions at 4 dB SNR for the “smaller PTA” and “larger PTA” subgroups (left panel) and the “better EC” and “worse EC” subgroups (right panel)**. Error bars show 95% confidence intervals. Black solid horizontal bars denote significant differences for the “smaller PTA” and “better EC” subgroups, gray dotted horizontal bars denote significant differences for the “larger PTA” and “worse EC” subgroups, and black dashed vertical bars denote significant differences among subgroups within a given HA condition (^*^p < 0.05, ^**^p < 0.01, ^****^p < 0.0001, ^*****^p < 0.00001). HA denotes HA condition.

To scrutinize the significant interactions between HA condition, PTA, and EC_PC_ we carried out series of planned contrasts on the data from 0 to 4 dB SNR. Effects were clearest at 4 dB SNR and are therefore shown in Figure 5. For both microphone settings, the “larger PTA” subgroup more strongly disliked inactive NR than the “smaller PTA” subgroup, whereas for strong NR the situation was reversed (all *p* < 0.05). Similarly, for the omnidirectional (but not the cardioid) microphone setting the “worse EC” subgroup more strongly disliked inactive NR than the “better EC” subgroup, whereas for strong NR the situation was reversed (both *p* < 0.05). At 0 dB SNR, the picture was very similar although the differences in mean preference between the “smaller PTA” and “larger PTA” subgroups were no longer significant at the 5% level for the DIR_off_NR_str_ and DIR_on_NR_off_ conditions (both *p* = 0.06). The same was true for the difference in mean preference between the “better EC” and “worse EC” subgroups for the DIR_off_NR_off_ condition (*p* = 0.08). Finally, it should be noted that whereas at 0 dB SNR all subgroups preferred moderate NR the most, at 4 dB SNR the “larger PTA” and “worse EC” subgroups tended to more strongly prefer strong NR. Nevertheless, because of the considerable inter-individual variability in preference for strong NR, the mean scores for the moderate and strong NR settings did not differ statistically from each other (all *p* > 0.1).

Altogether, these results suggest that in terms of preference HA users with larger PTA fare better with stronger NR than HA users with smaller PTA irrespective of microphone mode. Similarly, they suggest that HA users with poorer EC performance also fare better with stronger NR than HA users with better EC performance, but only in combination with the omnidirectional setting.

## Discussion

The current study had four main aims: (1) to confirm the previously observed association between RS and preferred NR setting, (2) to find out if there are similar associations with the DIS and EC measures, (3) to investigate if PTA, input SNR, and DIR also modulate preferred NR setting, and (4) to find out if preference and speech recognition show similar relations to PTA and the measures of executive function. Regarding the first aim, we saw no indications in the data from the current study that RS interacts with preferred NR setting. Regarding the second aim, DIS did not affect preference for the various HA conditions either, whereas EC_PC_ could partly account for the observed inter-individual variability. Regarding the third aim, we found larger PTA to be associated with weaker preference for inactive NR and stronger preference for strong NR, preference for strong NR to increase with input SNR, and DIR to weaken the association between EC_PC_ and preferred NR setting. Regarding the fourth aim, we observed that PTA and the measures of executive function interacted differentially with preference and speech recognition. In the following sections, we discuss these results in more detail.

### Effects of executive functions

As pointed out above, it is not uncommon to observe weak correlations among different measures of executive function, which was also the case in the current study (see Results). Presumably, this was at least partly because we had used *non-verbal* benchmarks for the *verbal* RS measure. We therefore had expected that these measures would give rise to different patterns of association with HA outcome, and this is also what we found.

In our previous study, listeners with shorter RS had preferred strong over moderate NR, whereas listeners with longer RS had not (see Introduction). However, our current study revealed no influence of RS on preferred NR strength (nor on preferred microphone setting). For the current study, we had deliberately recruited new participants. One reason for the divergent results across studies concerning the influence of RS could therefore be that the salient characteristics were not sufficiently pronounced in the cohort tested this time—perhaps because we had screened potential candidates more rigorously. In fact, however, the RS scores of the cohorts from the previous and current study were very similar (mean RS scores: 38.2 vs. 36.0%-correct; coefficients of variation: 0.27 vs. 0.27), thereby ruling out such an explanation. Another reason for the conflicting results could be random sampling variation. In principle, it is possible that preference for NR strength is a very individual trait that is not easily captured by a given measure of executive function. If this were the case, it would not be possible to assess the influence of executive function on the NR strength preferred by elderly HA users reliably based on a few samples of that population.

Apart from RS, DIS was also unrelated to preference for HA condition. To recapitulate, we had included DIS as a non-verbal benchmark for the RS measure indexing different executive functions (i.e., selective attention and inhibition). Incidentally, the spread in the DIS data was notably larger (coefficient of variation = 1.6) than in the RS data. In spite of this, DIS failed to account for any of the inter-individual variability in our preference scores. We therefore conclude that this measure was not sensitive to the executive processes driving preference for the HA conditions tested here.

In contrast to the other measures of executive function, EC_PC_ was associated with preference for our HA conditions. This was despite the fact that the spread in the EC_PC_ data (coefficient of variation = 0.22) was smaller than in the DIS and RS datasets. Our motivation for including EC was to have another non-verbal benchmark for RS indexing a wider range of executive functions than DIS (i.e., working memory, mental flexibility, selective attention, and inhibition). At present, it is unclear why precisely EC_PC_ could explain some of the variability in our preference scores. We speculate that because of the relatively broad spectrum of executive functions it taps into it was in a better position to capture the executive processes governing our listeners' preference judgments. Future research should try to identify the precise factors driving the observed association, ideally with the help of a new cohort of HA users.

### Effects of hearing loss

Regarding hearing loss, our earlier study had indicated that listeners with larger PTA prefer stronger NR than listeners with smaller PTA (see Introduction), and the results from the current study were consistent with this.

Only a couple of studies seem to have investigated the influence of PTA on preferred NR setting so far. In one study, five single- or multichannel NR schemes were tested, including the binaural coherence-based algorithm tested by us (Luts et al., 2010). Groups of listeners with normal hearing (ages 16–52), flat hearing losses (ages 22–79), and sloping hearing losses (ages 51–80) participated. Outcome measures included speech recognition and overall preference. For most NR schemes, the changes in outcome were very similar across groups, suggesting a negligible influence of PTA. For the binaural coherence-based algorithm, however, a significant effect of listener group was observed. That is, whereas the two hearing-impaired groups preferred this type of NR over no processing, the normal-hearing listeners did not. In another study, Houben et al. (2012) investigated preferred NR strength for two single-channel algorithms. Ten normal-hearing listeners (ages 21–31) and seven listeners with sloping hearing losses in the mild to severe range (ages 25–61) participated. For both groups, considerable inter-individual differences in preferred NR strength were observed. Also, their data overlapped considerably, resulting in a non-significant group effect. However, due to the small sample size and the fact that no attempt was made to control for any other factors that may affect HA outcome (e.g., age or executive functions), this result is not particularly surprising.

In summary, the influence of PTA that we observed was consistent with our previous data and, broadly speaking, also the results of Luts et al. (2010). The fact that Luts et al. did not find a corresponding group difference for any of their other NR schemes raises the question of whether the observed influence of PTA only pertains to the binaural coherence-based algorithm tested here. This should be addressed by future research.

### Effects of SNR and microphone directionality

Concerning the influence of SNR, our earlier study had indicated that preference for strong NR increases with input SNR (see Introduction), and the results from the current study were consistent with this. This dependency can be traced back to the fact that with higher input SNR the adverse effects of the NR processing (i.e., speech distortion) decreased while its positive effects (i.e., noise attenuation) increased, as confirmed by our technical analyses (see Tables 2, 3). Consequently, the benefit from strong NR increasingly outweighed its unwanted side effects. Based on this interpretation, one would expect even stronger preference for strong NR above 4 dB SNR. In actual fact, this is what we observed in our previous study, as part of which we had also collected preference judgments at 8 dB SNR (see Introduction). Interestingly, we did not observe any effects of PTA or the measures of executive function at −4 dB SNR. Previously, we had observed rather poor reproducibility for NR preference ratings at −4 dB SNR, whereas at 0 and especially 4 dB SNR reproducibility had been much better (Neher et al., 2014). Perhaps because speech distortion was greatest at −4 dB SNR participants were unsure about their preferences, thereby leading to no consistent associations with PTA or the measures of executive function.

Concerning the influence of DIR, we observed a clear preference for the cardioid over the omnidirectional setting. This is consistent with the finding of other researchers that DIR is preferred when noise is present and the signal of interest is in front of, and relatively near to, the listener (e.g., Walden et al., 2004, 2005). Furthermore, we had hypothesized that because directional preprocessing can reduce the amount of speech distortion caused by NR this might affect the influence of executive functions on preferred NR setting. Our technical analyses confirmed an improvement in speech quality due to DIR (see Table 3). Our perceptual analyses revealed that the observed association between EC_PC_ and preferred NR strength only applied to the HA conditions without DIR. Thus, these findings were consistent with our hypothesis. At first sight, they are also consistent with the idea that executive processes modulate susceptibility to HA distortion, as proposed by Lunner et al. (2009). According to their view, individual differences in working memory capacity determine listening success with specific types of HA technology. In particular, listeners with greater working memory capacity are thought to be better at segregating a target signal from any unwanted artifacts as they can deploy some of this capacity for explicit (as opposed to implicit or effortless) processing needed to match suboptimal input with phonologically based long-term representations in their mental lexicon (cf., Rönnberg, 2003; Rönnberg et al., 2008). Although this view is consistent with the results from a number of HA studies focusing on speech recognition outcomes (see Introduction), it seemingly disagrees with the effects apparent in our preference data. This is discussed further below.

### Overall preference vs. speech recognition

In HA research, preference judgments and speech recognition measurements commonly produce divergent data patterns (e.g., Walden et al., 2005; Brons et al., 2013; Jensen et al., 2013). In view of this as well as our earlier results (see Introduction), we had expected PTA and the measures of executive function to be differentially related to our speech and preference scores. To summarize, our analyses of the preference scores had suggested that HA users with larger PTA fare *better* with stronger (i.e., more aggressive) NR, whereas for listeners with smaller PTA the opposite holds true (at 0 and 4 dB SNR with and without DIR). Furthermore, they had suggested that listeners with worse EC_PC_ performance fare also *better* with stronger NR, whereas for listeners with better EC_PC_ performance the opposite holds true (at 0 and 4 dB SNR without DIR). Our analyses of the speech scores, on the other hand, had suggested that HA users with larger PTA fare slightly *worse* with stronger NR than HA users with smaller PTA (at −4 and 0 dB SNR with DIR). Furthermore, they had suggested that HA users with worse RS performance fare slightly *better* with strong NR than HA users with better RS performance (at 0 dB SNR without DIR).

Taken together, there appears to be some consistency across our preference and speech recognition results concerning the influence of executive functions (but not PTA) on response to our HA conditions. Recall, however, that we used different measures of executive function for the analyses of the two datasets. This was because we had found the (linguistically based) RS measure to be predictive of the speech but not the preference scores, while for the (non-verbal) EC measure the opposite was true. Broadly speaking, this pattern of results is consistent with previous reports of the strongest associations between verbal measures of executive function (in particular RS) and speech recognition (cf., Akeroyd, 2008).

Importantly, the associations with EC_PC_ and RS that we found were in disagreement with the literature finding that HA users with longer RS fare better with more aggressive HA settings and vice versa (see Introduction). Incidentally, even though statistically significant, the across-subgroup effects of RS (and PTA) in our speech scores were on the order of a few percentage points only. One could speculate that for a clear influence of executive functions on the speech recognition with different HA conditions to emerge listeners need to be confronted with more pronounced signal distortions such as those caused by frequency compression (cf., Arehart et al., 2013). Some support for this is available from a recent study of Keidser et al. (2013) concerned with individual differences in speech recognition benefit from DIR—a type of HA technology that is typically free from any distortions for the target direction (e.g., Dillon, 2012)—which failed to find a clear influence of executive functions (and PTA). In principle, it is also possible that different executive functions interact differentially with the signal changes caused by different HA algorithms.

In summary, the reported influence of executive functions on response to HA signal processing differs somewhat across HA outcomes and studies. Future research in this field should therefore ideally focus on trying to reconcile the findings from different studies.

### Implications for hearing aid fitting

The results from our study imply that moderate NR works well for the majority of elderly HA users, especially when applied in conjunction with DIR (see Figures 2, 4). They also show that HA users experience benefit from NR processing at positive SNRs (see Figure 4) where at least some HA manufacturers curtail the efficacy of their NR schemes (cf. Smeds et al., 2010). Furthermore, our results suggest that a notable proportion of elderly HA users prefer strong over moderate NR. Because strong NR may interfere with speech intelligibility, it is important to be able to identify candidates for strong NR reliably. Although our analyses had revealed that PTA and EC_PC_ can partly account for the inter-individual variability in preference for inactive and strong NR, their predictive power was limited (with unique *R*^2^ for PTA and EC_PC_ amounting to about 11 and 6.3%, respectively, at 0 and 4 dB SNR). In addition, mean preference scores for the various subgroups did not differ statistically across the moderate and strong NR settings (see Figure 5). A relevant question therefore is whether the combined predictive power of PTA and EC_PC_ is sufficiently large to allow determining candidature for moderate or strong NR. To address this we performed a supplementary ANOVA for which we grouped PTA and EC_PC_ into a single H±EC± factor (akin to the H±C± factor we had used previously). Results showed that the H–EC– subgroup significantly preferred DIR_off_NR_str_ over DIR_off_NR_mod_ at 4 dB SNR (mean preference scores: 0.55 vs. 0.44 scale points; *p* = 0.029). Otherwise no differences in preference for strong over moderate NR were observable (all *p* > 0.16).

Altogether, our results indicate the basic potential of individualizing NR based on PTA and (to a lesser extent) executive functions. Furthermore, they point toward a need for alternative diagnostic measures that can capture more of the variability in preference for different NR settings, and current work in our laboratory is concerned with this issue.

### Conflict of interest statement

The research reported in this article was co-funded by Siemens Audiologische Technik, Erlangen, Germany. However, the contents represent the work and private views of the author only.
